# Differential Culturability of *Mycobacterium tuberculosis* in Culture-Negative Sputum of Patients With Pulmonary Tuberculosis and in a Simulated Model of Dormancy

**DOI:** 10.3389/fmicb.2019.02381

**Published:** 2019-10-23

**Authors:** Azger Dusthackeer, Magizhaveni Balasubramanian, Govindarajan Shanmugam, Shanmuga Priya, Christy Rosaline Nirmal, Rajadas Sam Ebenezer, Angayarkanni Balasubramanian, Rajesh Kumar Mondal, Kannan Thiruvenkadam, A. K. Hemanth Kumar, Geetha Ramachandran, Selvakumar Subbian

**Affiliations:** ^1^National Institute for Research in Tuberculosis, Chennai, India; ^2^National Institute of Epidemiology, Chennai, India; ^3^Public Health Research Institute, New Jersey Medical School, Rutgers University, Newark, NJ, United States

**Keywords:** tuberculosis, bacteriological diagnosis, resuscitation, *Mycobacteirum tuberculosis*, clinical isolate organism, sputum samples, dormancy

## Abstract

Tuberculosis (TB) remains a leading killer among infectious diseases of humans worldwide. Delayed diagnosis is a crucial problem in global TB control programs. Bacteriological methods currently used to diagnose TB in endemic countries take up to 8 weeks, which poses a significant delay in starting antibiotic therapy. The presence of a heterogeneous population of *Mycobacterium tuberculosis*, the causative agent of TB, is among the reasons for delayed diagnosis by bacteriological methods. Previously, it has been shown that mycobacterial resuscitation-promoting factors (RPFs), a family of proteins secreted by actively growing bacteria into the media, are capable of activating the growth of dormant bacteria, thus enhancing the detection of bacilli in the sputum of confirmed TB cases. However, the variability in bacterial resuscitation by RPF in the sputum of suspected pulmonary TB cases that showed differential smear and/or culture positivity during diagnosis has not been fully explored. Here, we report the presence of non-replicating bacteria in the sputum of suspected TB cases that show differential growth response to RPF treatment. Using crude and recombinant RPF treatment, we show improved sensitivity and reduced time to detect bacilli in the sputum samples of smear-positive/culture-negative or smear-negative/culture-negative cases. We also report the phenotypic heterogeneity in the RPF responsiveness among Mtb strains using an *in vitro* dormancy model. Our findings have implications for improving the bacteriological diagnostic modalities currently used to diagnose TB in endemic countries.

## Introduction

Tuberculosis (TB) caused by *Mycobacterium tuberculosis* (Mtb) is a leading killer among infectious diseases that accounts for 1.5 million deaths and about 10 million new cases worldwide in 2017. Nearly 1.7 billion (23%) of the global population have been estimated to harbor asymptomatic latent Mtb infection (LTBI)^1^. One of the key characteristics that make Mtb a successful intracellular pathogen is its ability to survive amid various stresses, including hypoxia, reactive oxygen, and nitrogen species (ROS and RNS) and nutrition starvation that prevails in the infected host (Voskuil et al., 2011). Besides, Mtb can establish a non-replicating dormancy state and persist in host tissues for prolonged periods without causing symptomatic disease (Gomez and McKinney, 2004; Gengenbacher and Kaufmann, 2012). Importantly, individuals with LTBI, harboring quiescent Mtb population, can resume active bacterial growth to develop symptomatic TB if/when the host immunity wanes (Magombedze et al., 2013; Peddireddy et al., 2017). However, the host and bacterial factors responsible for reactivation of dormant Mtb upon immune-suppressing host conditions are not fully understood.

The efficiency of the standard multidrug antibiotic regimen currently used for TB treatment is closely linked with the nature of Mtb in the infected host. Clinical and experimental studies have demonstrated the presence of various Mtb phenotypes to co-exist in the same sample than it was previously thought (Mukamolova et al., 2010; Ayrapetyan et al., 2015). While actively replicating bacilli are killed rapidly and effectively by first-line drugs such as isoniazid, the dormant form of Mtb is seldom eliminated by the current antibiotic regimen (Deb et al., 2009). The presence of dormant bacilli that are resilient to killing by antibiotics is one of the reasons for the prolonged duration (minimum of 6 months) of current treatment (Zhang, 2004). The ability of dormant Mtb to persist in a non-replicating state that is phenotypically tolerant to drugs is also a significant impediment in the diagnosis and treatment of LTBI cases; at present, there are no growth-based tests available to measure the burden of dormant bacilli in clinical samples (Cardona and Ruiz-Manzano, 2004). Although the presence of dormant, non-replicating bacteria has been shown in the sputum of patients with active pulmonary TB that showed positive culture results, these bacillary populations do not grow well on standard agar media used for diagnosis. Moreover, little is known about the physiology and phenotypic nature of Mtb persisting during LTBI *in vivo*.

Resuscitation-promoting factors (RPFs), a group of five proteins (Rpf A–E) secreted by actively replicating virulent Mtb into the culture media, have been demonstrated to reactive at growth of dormant bacteria *in vitro* and in the sputum of patients with TB (Gupta and Srivastava, 2012). Although sputum has traditionally been thought to contain exclusively actively replicating Mtb, bacterial transcript analyses disprove this notion (Garton et al., 2008). For example, Mukamolova et al. (2010) have shown that smear-positive sputum samples from TB patients are dominated by the presence of Mtb that is mostly non-cultivable by conventional, standard bacteriological methods used in diagnostic settings. Recently, Kana et al. have shown the presence of differentially cultivable tubercle bacilli (DCTB) in the sputum of culture-positive pulmonary TB patients. These samples had bacteria that showed heterogeneity in their response to treatment with RPF-containing culture filtrate (Chengalroyen et al., 2016).

Moreover, the RPF dependency of Mtb was lost after the bacteria were isolated from the patient sputum. Interestingly, during TB chemotherapy, the proportion of RPF-dependent Mtb increased relative to the actively growing bacterial population that form colonies on standard growth media (Mukamolova et al., 2010). Thus, change in the number of non-cultivable Mtb during and at the end of chemotherapy depends on the ability of this population to become dormant (Nathan and Barry, 2015). These findings suggest that the sensitivity of existing culture-based techniques to detect Mtb in pulmonary samples can be considerably improved with RPF treatment. However, the ability of RPF-containing media to resuscitate Mtb in the sputum samples of suspected TB cases that are smear-positive and culture-negative or smear and culture-negative status remains unclear. This insufficiency in current bacteriological tools to efficiently diagnose cases with subclinical and incipient forms of TB has a high impact on global TB elimination goals, since they pose additional challenges on diagnosis and treatment, compared to smear- and/or culture-positive cases (Garcia-Basteiro et al., 2019).

Previously, using a diagnostic luciferase reporter phage assay that can detect non-replicating bacilli in patient sputum, we have identified 30 additional positives samples, which failed to grow on standard Lowenstein–Jensen (LJ) agar medium (Dusthackeer et al., 2012). The presence of viable bacilli in these samples was confirmed by reverse transcriptase-PCR for the *Mtb* 16S rRNA gene, indicating that either the improved sensitivity of the assay to detect actively growing bacilli or its ability to detect non-replicating persistor bacilli ultimately increased the diagnostic potential of culture-based assays. In this study, we provide more evidence for the presence of non-replicating persistors in the sputum specimen of suspected TB cases that showed negative result in the conventional microbiological diagnosis. Using RPF-containing culture filtrates of Mtb to treat sputum samples of suspected TB cases, we have improved the sensitivity and rapidity of standard culture-based diagnosis. We also report the phenotypic heterogeneity in RPF-responsive Mtb *in vitro* using Wayne’s dormancy model.

## Materials and Methods

### Clinical Specimen and Ethics Statement

Previous studies have evaluated the presence of RPF-dependent Mtb in smear and/or culture-positive sputum samples from active TB patients (Mukamolova et al., 2010; Chengalroyen et al., 2016). In our study, we wanted to investigate the presence of RPF-dependent Mtb in suspected TB cases from local patients in TB-endemic South Indian region (Chennai; coordinates: 13.04°N 80.17°E). The sputum samples from these patients varied in their microbiologic diagnosis (i.e., smear-positive/culture-negative, smear-negative/culture-negative, and smear-positive/culture-positive). A total of 421 sputum specimens, collected from consecutive adult patients (two samples per patient, see below) suspected of TB were used in this study. Of these, 251 were smear- and culture-negative, 17 were smear-negative but culture-positive, 55 were smear- and culture-positive, and 98 were smear-positive but culture-negative (Supplementary Figure S1). Sputum samples were collected at the baseline (minimum of 5 ml, before the start of treatment) from suspected TB patients. These patients were newly diagnosed of suspected TB and were about to start their standard antibiotic therapy. All sputum samples were collected in a routine clinical setting at the National Institute for Research on Tuberculosis. Expectorated sputum samples were collected using standardized procedures and processed in a microbiology laboratory under sterile conditions. No follow-up sputum samples were collected from these patients.

From each patient, two sputum samples were collected; one collected at home and the other collected at the clinic. Both samples were separately processed by modified Petroff’s method, as mentioned in the manuscript. There was no discrepancy in the results, such as smear positivity rating or culture results obtained between these two sputum samples for any of the patients. We used the sputum collected in the clinic for consistency in collection and processing methodology between patients. The sterility of the collection container and proper collection of samples were ensured by medical personnel. The sputum samples were stored at −80°C until further processing. Sputum samples were processed by modified Petroff’s method, and smear positivity was assessed by sputum microscopy using auramine phenol staining (Verma et al., 2013; Datta et al., 2019). The total sample size was estimated at 5% level of the significance at 80% power with the design effect of two. Also, the 10% error rate was added to compensate for the follow-up/estimation error. Informed consent was obtained from the patients, and the Institutional Ethical Committee of the National Institute for Research on Tuberculosis, a body of Indian Council of Medical Research approved the sample collection and downstream application procedures.

### Bacterial Strains

Strains of *M. tuberculosis* (H37Rv, H37Ra, Erdman, DRBL2, MTB01, MTB02), *Mycobacterium smegmatis*, and *Escherichia coli* DH5α were grown and maintained as per standard procedures in LJ slants, Middlebrook 7H9 broth, and Middlebrook 7H11 agar media supplemented with oleic acid dextrose and catalase enrichment (OADC) (BD Biosciences) and glycerol or in Luria-Bertani (LB) media (for *E. coli*)^2^. In all the experiments, bacterial cultures at the mid-log phase (OD_600_ = 0.6) were used unless stated otherwise.

### Preparation of Crude RPF Containing Media

Crude RPF-containing culture filtrate from bacterial cultures was prepared as described previously (Kana et al., 2008). Briefly, wild type and laboratory strains of *M. tuberculosis* and *M. smegmatis* were grown in Middlebrook 7H9 media supplemented with OADC and *E. coli* were grown in LB media to an OD_600_ = 0.4. Bacterial cultures were centrifuged at 12,000 × *g* for 20 min at 10°C, and the supernatant was filtered through a 0.2-μm membrane filter and immediately diluted with an equal volume of 7H9 broth. The bacterial cultures were treated with RPF protein at a 1:10 ratio. The culture was plated on Middlebrook 7H11 agar media. At 7 days post-treatment, the colony-forming units (CFU) were enumerated.

### Effect of RPF on Culture Sensitivity

The impact of RPF in increasing the sensitivity of Mtb detection and the presence of non-replicating persistors was tested on the sputum samples of suspected TB patients. A total of 268 samples, including smear/culture-negative (*n* = 251) and smear-negative/culture-positive (*n* = 17) sputum samples, were used to determine the presence of resuscitable Mtb (RCs). Bacterial growth induction was confirmed by both the increase in the optical density (OD_600_) of broth culture and growth of RC on Middlebrook 7H11 agar plates. The results for each sample were compared to their corresponding diagnostic smear and/or culture status from the clinic.

One set of processed sputum sediment of 0.9 ml from smear-positive, culture-negative patients was treated with 0.1 ml of RPF for the resuscitation of Mtb and inoculated onto LJ slants, incubated at 37°C, and observed at 7-day intervals for the appearance of Mtb colonies for 8 weeks. Control samples were treated with sterile 7H9 broth, plated on LJ slants, and kept at similar growth conditions as above. For liquid culture, the minimum probable number (MPN) assay was used as described previously (Chengalroyen et al., 2016). Briefly, smear-negative and culture-negative sputum specimens were treated with crude RPF at 1:9 ratio and incubated at 37°C for 4 weeks. Another aliquot of the same sample was treated with sterile 7H9 broth as a control. Bacterial growth was measured by an increase in OD_600_ at every 24 h using a spectrophotometer. The possibility of contamination of the cultures was screened by spotting 5 μl of the culture onto Brain Heart Infusion agar plates. The presence of non-replicating persistors and their sensitivity to antibiotics were determined by comparing the number of bacterial CFU between RPF-treated and untreated samples.

### Effect of RPF on Time to Detect (TTD) Mtb in Clinical Samples

To test whether RPF can reduce the TTD Mtb, we analyzed 55 smear- and culture-positive sputum specimens. The sputum-smear gradation of smear-positive/culture-positive samples used to evaluate TTD Mtb is presented in Supplementary Table S1. Each sputum sample was divided into two aliquots and treated with either crude RPF or sterile 7H9 broth, respectively, and plated on 7H11 agar media as described above. The plates were observed periodically for 8 weeks for bacterial growth, and the difference in the time taken for the appearance of Mtb colonies was recorded in each case.

### Comparative Assessment of Resuscitation Potential of Crude and Recombinant RPF

Recombinant RPF (rRPF) protein from Mtb that was heterologously expressed using an *E. coli* system was a kind gift from Tom H. Ottenhoff of Leiden University Medical Center, Netherlands (Commandeur et al., 2011). The rRPF contains RPF-A (Rv0867c) and RPF-D (Rv2389c) proteins. Details about the cloning and characterization of the rRPF proteins have been published previously (Commandeur et al., 2011). These two RPFs (out of five) were selected since they showed the highest recognition by TB patient samples in a cross-sectional cohort study (Commandeur et al., 2011). The rRPF was transported under cold-chain conditions and stored at −20°C till further use. The efficiency of rRPF (10 pmol) in resuscitating dormant bacteria was compared with crude RPF extracted directly from the *Mtb* cultures. A total of 98 smear-positive, culture-negative samples were split into three aliquots, and each was treated with sterile 7H9 broth (control) or crude RPF or rRPF, respectively. Treated samples were inoculated onto LJ slants, incubated at 37°C, and observed till 8 weeks for Mtb colonies. The number of Mtb colonies and yield of positivity were noted and compared between the groups.

### Effect of RPF on Mtb Dormancy *in vitro*

The potential of RPF in resuscitating Mtb from dormancy to metabolically active form was tested using an *in vitro* anaerobic culture system developed by Wayne and Hayes (1996). In brief, two clinical Mtb isolates, MTB01 and MTB02, were inoculated at a final OD_600_ = 0.1 into 7H9 broth supplemented with OADC and glycerol in an airtight container and incubated at 37°C with very gentle stirring. As reported previously, at 45 days post-inoculation, a hypoxic, non-replicating persistent stage (NRP) was achieved under these conditions, as indicated by discoloration of methylene blue indicator (Supplementary Figure S2; Wayne and Hayes, 1996). At this state, Mtb adapts to a dormant phenotype. This state of metabolically inactive dormant condition was also confirmed by spotting the bacterial culture onto 7H11 agar media supplemented with OADC and glycerol. The quiescent bacterial cultures were treated with either sterile 7H9 broth (control) or crude RPF extracted from pathogenic Mtb (H37Rv, Erdmann), non-pathogenic mycobacteria (H37Ra, *M. smegmatis*), or *E. coli* DH5α as mentioned above. The culture exposed to RPF or control broth was serially diluted and spotted onto 7H11 agar plates, incubated at 37°C, and observed regularly for up to 8 weeks for the appearance of Mtb colonies.

### Statistical Analysis

Statistical significance for pairwise comparisons was performed using paired Student’s *t* test, and multiple comparisons were calculated by one-way ANOVA using GraphPad Prism. *P* value < 0.05 was considered statistically significant.

## Results

### Crude RPF Increases the Sensitivity of Liquid Culture

**FIGURE 1:**
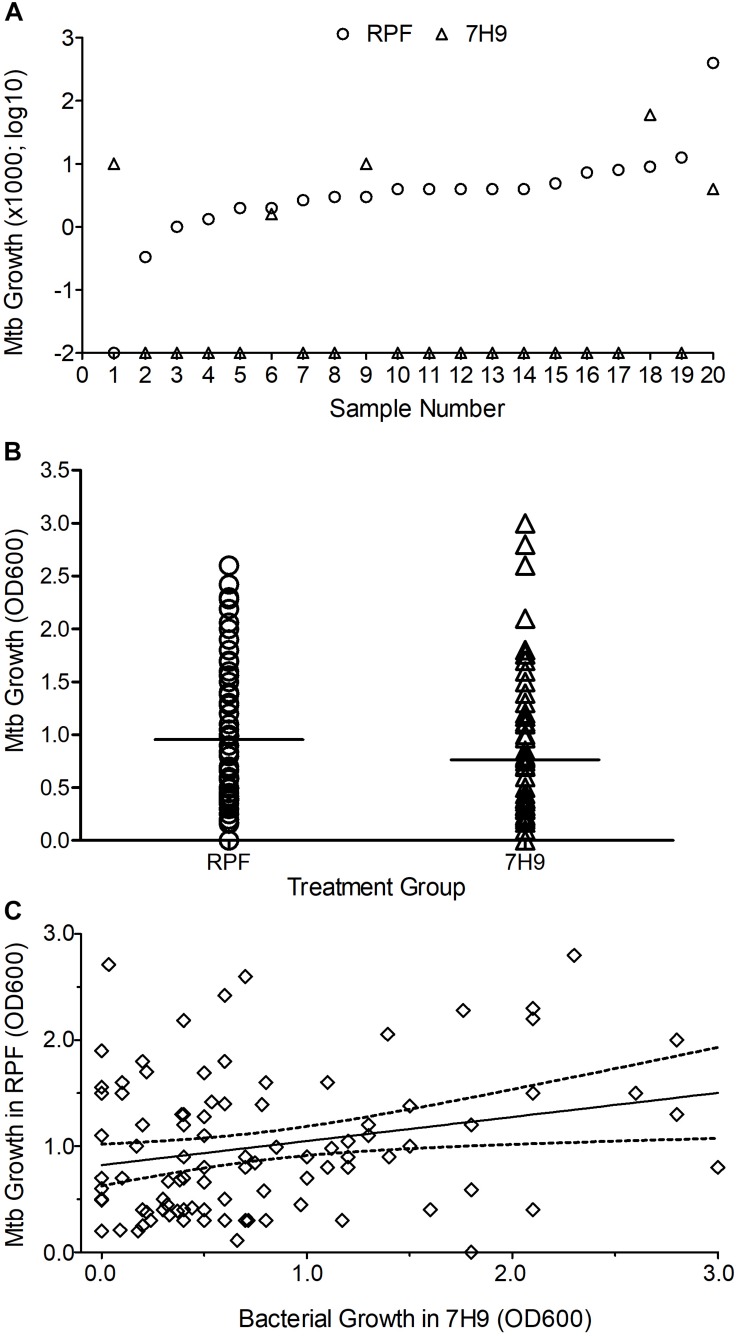
Effect of crude RPF on Mtb growth. **(A)** Bacterial CFU obtained from sputum samples treated with crude RPF or placebo (7H9). The difference in CFU between RPF (23.9 CFU ± 19.8) and 7H9 (4.3 ± 3.0) is not statistically significant as determined by paired Student’s *t* test (*P* = 0.34). **(B)** Bacterial growth in the sputum samples treated with crude RPF or placebo (7H9) as determined by OD_600_. The difference in OD_600_ between RPF (0.96 ± 0.06) and 7H9 (0.76 ± 0.08) is statistically significant as determined by paired Student’s *t* test (*P* = 0.036). **(C)** Linear regression analysis for the correlation significance of Mtb growth in crude RPF versus 7H9 broth. Treatment with RPF significantly improved bacterial recovery (*P* = 0.016).

In the agar plating method, among the crude RPF-treated samples, 20 out of 268 (7.5%) yielded bacterial CFU and were positive for RCs. These samples were previously classified as negative by conventional smear/culture-based diagnostic methods. Among these 20 samples that showed positive CFU with RPF, treatment with sterile 7H9 broth (placebo) resulted in Mtb CFU, and positive RCs for only five samples (25%) (Figure 1A). Thus, the treatment of smear/culture-negative sputum with crude RPF can improve case detection by about 75%. In addition, about 2% of smear/culture-negative sputum spontaneously reverts to bacterial growth, as shown in samples treated with placebo broth. Overall, crude RPF treatment resulted in nearly a four-fold higher number of positive case detection among smear/culture-negative sputum samples.

In the MPN assay that is based on bacterial growth in liquid media, a significant (*P* = 0.036) increase in the number of positive samples was noted between crude RPF-treated and placebo broth-treated samples (86 vs. 77 out of total 268 samples) (Figure 1B). We performed a linear regression analysis to determine the correlation significance between samples treated with RPF and 7H9 broth. As shown in Figure 1C, bacterial growth in samples treated with RPF was significantly high, compared to the same samples treated with 7H9 media (*P* = 0.0163), which clearly shows that RPF significantly induces bacterial growth (Figure 1C). The standard deviation from the average is low for RPF-treated, compared to 7H9-treated samples, which indicates the consistency of RPF in resuscitating Mtb in the sample. The culture positivity in both liquid and solid media was further confirmed by acid-fast staining, and microscopy.

### Crude RPF Treatment Significantly Reduces the TTD Mtb in Clinical Specimens

**FIGURE 2:**
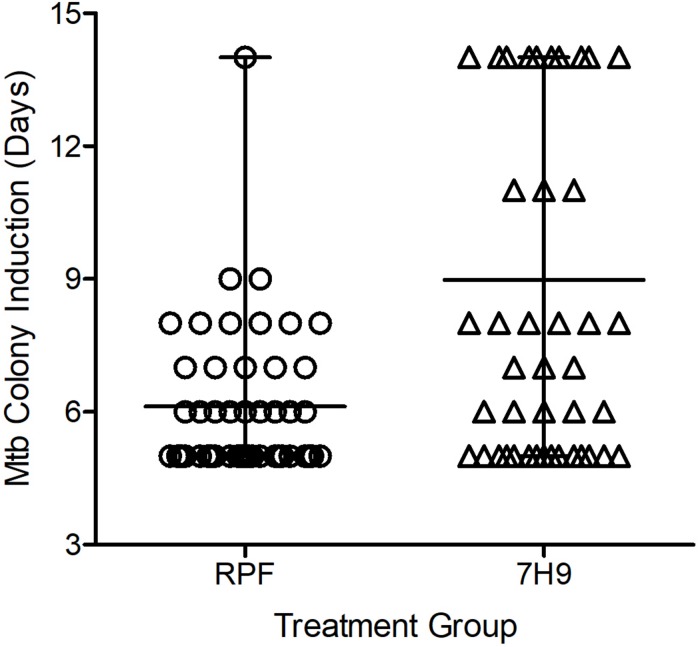
Effect of crude RPF on time to detect (TTD) Mtb from sputum samples of smear-positive and culture-positive TB cases. The TTD analysis shows a significant reduction in time when samples are treated with crude RPF, compared to placebo (7H9) as analyzed by paired Student’s *t* test (*P* < 0.001).

The possibility of reducing the TTD Mtb in clinical specimens by using crude RPF treatment was tested in 55 smear- and culture-positive sputum samples. The average time taken for positive Mtb detection was 6.1 days for RPF-treated samples, compared to an average of about 9 days for the 7H9 broth (placebo)-treated samples (*P* ≤ 0.001) (Figure 2). Therefore, treatment with crude RPF significantly reduced the TTD Mtb in the specimen as indicated by the appearance of colonies at least 3 days earlier than the placebo (7H9 media)-treated samples (Supplementary Figure S3).

### Crude RPF Is More Potent Than rRPF in Reactivating Mtb Growth

**FIGURE 3:**
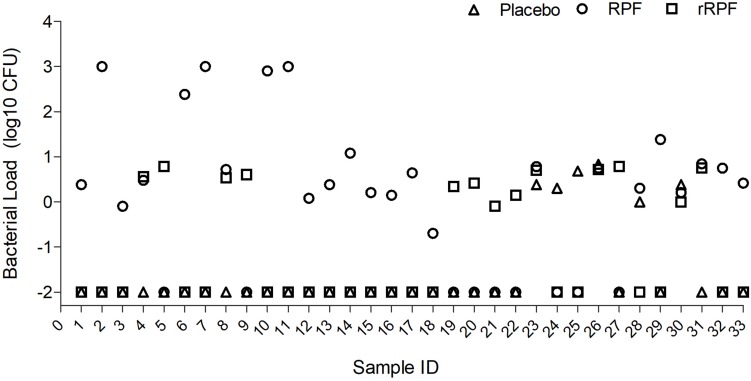
Effect of crude RPF and recombinant RPF proteins on Mtb growth. Sputum samples from 33 smear-negative/culture-negative TB patients were treated with placebo (H9) or crude RPF or recombinant RPF protein (rRPF). Treated samples were plated on 7H11 agar to enumerate bacterial CFU. The difference in bacterial CFU was statistically significant between H9 and RPF (*P* = 0.03), H9 and rRPF (*P* = 0.049), and RPF and rRPF (*P* = 0.032) as analyzed by one-way ANOVA with the *post hoc* test.

Among 98 smear-positive/culture-negative samples tested, treatment with crude RPF yielded positivity in 24 specimens (24.5%), while 6 samples (6.1%) showed positive results for Mtb when treated with placebo broth and 13 samples (13.2%) showed positivity for Mtb after treatment with rRPF. Four out of the six positives noticed in placebo-treated samples showed a higher culture yield when treated with crude RPF or rRPF. Most of the positives obtained by crude RPF treatment were reported negative by placebo broth treatment. Among the tested samples, the Mtb culture yield in crude RPF and rRPF treatment showed 4 log_10_ and 2 log_10_ higher positivity rates, respectively, compared to placebo broth treatment. Out of 13 positive cultures observed with rRPF treatment, seven yielded RCs only in the presence of rRPF and not with either crude RPF or placebo broth treatment. Similarly, 18 samples yielded positive RCs only in the presence of crude RPF, while two samples were positive exclusively for placebo broth treatment (Figure 3). These data suggest a higher positivity rate for samples treated with crude RPF, which is more effective in improving the sensitivity of Mtb detection in a clinical specimen, compared to treatment with recombinant RPF. However, some of the tested samples reactivated Mtb only in the presence of rRPF, while few other samples treated with placebo spontaneously revived Mtb growth. These observations suggest the complexity of Mtb growth stimulation during treatment with RPF, which remains to be determined.

### Effect of RPF From Different Bacterial Strains on Dormant Mtb

**FIGURE 4:**
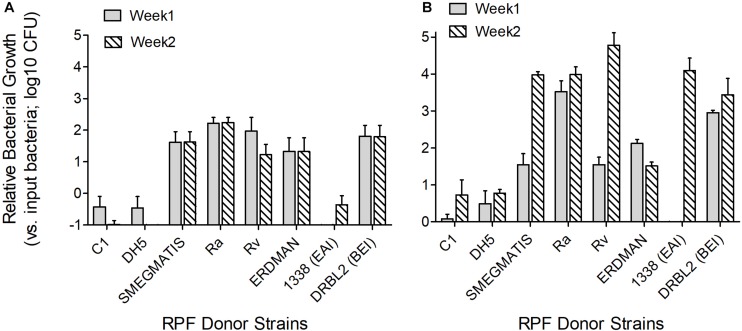
Effect of crude RPF isolated from different bacterial strains in resuscitating dormant clinical Mtb isolates. Clinical Mtb isolates MTB01 **(A)** and MTB02 **(B)** were grown to dormancy in an *in vitro* hypoxia model and treated with crude RPF isolated from *E. coli* (DH5a), *M. smegmatis* mc^2^155, Mtb H37Ra, Mtb H37Ra, Mtb 1338-EAI, Mtb DRBL2-BEI, and Mtb Erdmann strains or placebo (7H9 broth; C1). Treated Mtb culture was plated on 7H11 agar media to enumerate bacterial CFU. The data in **A** and **B**, except C1 and DH5, are significant; *P* < 0.05.

Crude RPF obtained from the cultures of different virulent Mtb strains, including H37Rv, DRBL2 (a clinical isolate of W-Beijing lineage), 1338 (a clinical strain of East African Indian lineage), Erdman, and avirulent mycobacteria such as H37Ra, *M. smegmatis*, as well as *E. coli* were tested for their potency to reactivate clinical Mtb strains (MTB01 and MTB02) grown *in vitro* to dormancy by oxygen depletion. Clinical Mtb isolates MTB01 and MTB02 were grown to dormancy and treated with the crude RPF obtained from various bacterial species (Figure 4). Results show heterogeneity in response between MTB01 and MTB02 in their ability to resuscitate from dormancy upon treatment with crude RPF (Figures 4A,B). The latter strain showed a robust response than the former, as measured by the number of cultivatable bacterial CFU. A clear difference in the number of reactivated dormant Mtb was noted with crude RPF from various bacterial cultures compared to 7H9 control broth (Figure 4). While crude RPF from *E. coli* failed to show any resuscitation of the tested Mtb strains, the crude RPF from mycobacteria, including H37Ra, H37Rv, W-Beijing, *M. smegmatis*, and Erdmann strains showed significant resuscitation of more number of bacilli, leading to an increased number of Mtb CFU from the sample. Thus, it appears that different mycobacterial strains/species, irrespective of their virulence potential, are capable of resuscitating dormant Mtb *in vitro*.

## Discussion

Tuberculosis is a disease of global importance. About 90% of Mtb-infected individuals are asymptomatic (latent) and harbor the bacteria in a dormant form. These cases have a 5–10% lifetime risk for reactivation to symptomatic TB (Gomez and McKinney, 2004; Gengenbacher and Kaufmann, 2012). Although both host- and bacteria-derived factors are implicated in the establishment of latency, the mechanisms underlying reactivation of dormant Mtb *in vivo* and the conversion of latent infection to active TB remains poorly understood (Kana and Mizrahi, 2010). RPFs, a family of muralytic enzymes, secreted by actively growing mycobacteria, are among such factors that are found to reactivate/resuscitate growth of dormant bacilli, which grow poorly or do not grow at all in conventional growth media that supports growth of actively replicating bacteria (Mukamolova et al., 1998, 2002; Kana and Mizrahi, 2010). Sputum specimens of patients with active TB are reported to harbor a heterogeneous population of metabolically different classes of Mtb, ranging from fully replicating to non-replicating persisters (Garton et al., 2008). In clinical specimens of patients with active pulmonary TB, the presence of Mtb population with quiescent metabolic stages was revealed upon treatment of the specimen with RPF-containing media (Mukamolova et al., 2010; Chengalroyen et al., 2016). Further, CF treatment of smear-positive sputum specimens from TB patients increased the number of bacterial CFU counts, compared to no-CF treated samples. Moreover, the resuscitating potential was lost when CF from the Mtb strain mutant for RPF genes was used to treat the sputum samples.

Similarly, biological samples, other than sputum, from patients with extrapulmonary TB were shown to contain differentially culturable Mtb by their ability to grow only in the presence of RPF-containing culture filtrate (Mukamolova et al., 2010; O’Connor et al., 2015). These results highlight the essentiality of functional RPF for the resuscitation of Mtb in clinical samples (Mukamolova et al., 2010; Chengalroyen et al., 2016).

In the present study, we have tested sputum specimens from patients with clinical features suspected for pulmonary TB; these specimens were mostly negative for Mtb in smear and culture tests, two key bacteriological diagnostic methods widely used in endemic countries. We used RPF-containing culture filtrate (CF) from actively growing mycobacteria or recombinant RPF to treat sputum specimens. Results clearly show improved detection of viable but non-culturable (VBNC) Mtb, based on growth in liquid media (measured by OD_600_) and growth of viable bacilli (viable cells – VC) on conventional agar media as described previously (Dusthackeer et al., 2012). In the current study, we were able to detect Mtb in about 75% more of several smear/culture-negative samples from patients who completed anti-TB therapy; these samples were declared negative by conventional microbiological diagnoses, such as smear microscopy and culture (unpublished results). Thus, our results suggest that even after successful completion of antibiotic treatment for TB, residual Mtb can persist without forming colonies on conventional microbiologic media. This leads not only to the underestimation of bacteriological cure rates among treated patients, but the persisting bacilli can cause reactivation of disease and contribute to the generation of drug resistance. Our findings are consistent with a study by Chengalroyen et al. that reports about 19% of sputum samples from TB patients with or without HIV co-infection to be dependent on culture filtrate (CF) (Chengalroyen et al., 2016). Another study tested the effect of RPF on sputum samples from TB patients before antibiotic treatment. Results of that study show that about 95% of bacteria were able to grow only in the presence of RPF-containing CF (Mukamolova et al., 2010), suggesting that the presence of RPF-dependent Mtb is frequent in a clinical specimen.

Our finding that a fraction of the NRP population of Mtb in clinical specimen responds to RPF treatment has clinical implications. Thus, in this study about 25% of the patients, whose specimen revealed the presence of RPF-dependent NRPs, either relapsed to active disease or met with treatment failure, including two patients who died of treatment-associated complications (unpublished results). Importantly, these two patients completed the full course of antibiotic treatment and showed negative-culture conversion, which was indicative of a good clinical prognosis. However, in the sputum samples of these patients, the presence of the RPF-dependent NRP bacterial population was evident from our assays. Therefore, we predict that the presence of such RPF-dependent NRP is responsible for disease relapse in these patients. Taken together, the presence of resuscitable/viable Mtb even after successful completion of treatment provides a piece of crucial evidence for extending the current treatment duration and for testing the drug susceptibility pattern of infecting Mtb.

In our studies, the differential growth of Mtb in a clinical specimen was more prominent in the presence of crude RPF than treatment with a cocktail of purified, recombinant RPF. Previous reports show that both recombinant RPF and the culture filtrate supernatant of young cultures of *M. tuberculosis* were able to produce increased growth of viable cells among the cells maintained in the stationary phase of growth (Shleeva et al., 2002; Wu et al., 2008; Huang et al., 2014). These studies also show reduced TTD Mtb when recombinant RPFs were added individually to mycobacteria growth indicator tube (MGIT)-inoculated cultures and from sputum specimens. In addition, Mukamolova et al. (2010) reported the potential of the recombinant RPF proteins to induce the growth of Mtb maintained in the stationary phase of growth in a genus-specific manner, irrespective of the species of *Mycobacterium* [*M. luteus*, *M. smegmatis*, and *M. bovis* (BCG)]. These studies suggest that RPFs do not mimic the role of just being a growth supplement or nutrient; instead, these proteins play a vital mechanistic role as a growth stimulant, based on the requirement of picomolar level concentration in the medium to reactivate bacterial growth. Moreover, Turapov et al. concluded that the potential of both recombinant RPF and the culture filtrate supernatant are equivocal; although reports from other groups contradicted this observation (Mukamolova et al., 2010; Chengalroyen et al., 2016). Using the CF of quintuple Mtb mutant for RPF (rpfA–D), Chengalroyen et al. (2016) showed resuscitation of non-replicating Mtb, suggesting that RPF can be dispensable to a certain extent in resuscitating dormant Mtb. This could be due to the presence of other Mtb-derived factors, including mucopeptides and lipids, in the crude RPF.

In this study, we have not evaluated the effect of RPF on the diagnostic potential of MGIT. However, we have used the same components of MGIT assay in our culture conditions (i.e., using 7H9 media base supplemented with OADC and antibiotics). Therefore, our observations could be relatable and extended to MGIT conditions. Nonetheless, this claim warrants further evaluation with actual experiments to test the beneficial effect of RPF on MGIT assays, which will be the focus of our future research.

## Conclusion

In conclusion, we demonstrate the presence of viable Mtb in the clinical specimen of patients with pulmonary TB who had negative results in conventional microbiological tests. We show that crude RPF, as well as recombinant RPF proteins, are capable of resuscitating dormant Mtb and improve the bacillary load as well as reduce the duration to detect Mtb in a clinical specimen. The mechanistic basis for the heterogeneity among Mtb in their response to RPF needs further investigation. Our findings have clinical implications since the treatment of patient sputum samples with RPFs has the potential to improve the current microbiological diagnosis of TB.

## Data Availability Statement

All datasets generated for this study are included in the manuscript/Supplementary Files. The raw data is available upon request to AD.

## Ethics Statement

The studies involving human participants were reviewed and approved by Institutional Ethical Committee of the National Institute for Research on Tuberculosis, a body of Indian Council of Medical Research. The patients/participants provided their written informed consent to participate in this study.

## Author Contributions

AD and SS conceived the concept, designed the experimental studies, wrote and edited the manuscript. AD, MB, GS, SP, and AB performed the experiments. SS, KT, AD, CN, RS, RM, and KT analyzed the data. AH and GR assisted with sample procurement and documentation. All authors read and agreed to publish.

## Conflict of Interest

The authors declare that the research was conducted in the absence of any commercial or financial relationships that could be construed as a potential conflict of interest.

## Footnotes

1 https://www.who.int/tb/publications/factsheets/en/

2 http://www.nirt.res.in/pdf/bact/SOP.pdf

## Supplementary Material

FIGURE S1 Flow chart showing the type and number of samples used for different assays in this study.

FIGURE S2 Establishment of non-replicating persistence stage of Mtb. Actively growing Mtb culture was sub-cultured into fresh media containing methylene blue, an indicator dye for oxygen tension **(A)** that de-colorizes as the culture becomes hypoxic **(B)**. Representative vials are shown either immediately after Mtb inoculation (blue color; **A**) and after the establishment of hypoxia (no color; **B**).

FIGURE S3 Colony formation by Mtb treated with crude RPF or placebo. Dormant cultures of wild type Mtb H37Rv (a laboratory strain) and representative clinical isolates (2474 and 2572) were treated with crude RPF or 7H9 broth for day 3 days **(A)** or 45 days **(B)** and serial dilutions were plated on 7H11 agar media for colonies to appear. Visible colonies were noted on day-7 post-inoculation for samples treated with RPF **(A)**. Prominent colony formation was observed on day-21 post-inoculation for samples treated with control 7H9 broth **(B)**.

TABLE S1 Smear gradation of samples used in this study.
