# Supplementary material for: Differential Culturability of Mycobacterium tuberculosis in Culture-Negative Sputum of Patients With Pulmonary Tuberculosis and in a Simulated Model of Dormancy
Source: Front Microbiol. 2019 Oct 23;10:2381. doi: 10.3389/fmicb.2019.02381 (PMC6842972; doi:10.3389/fmicb.2019.02381)
Supplement: FIGURE S1 — Flow chart showing the type and number of samples used for different assays in this study. [file Data_Sheet_1.PDF]

Supplementary Table-1: The WHO and IUATLD grading scale for AFB positivity of clinical specimens used in this study

Reporting scale	*Sample size (n) and % positivity* [a]	*AFB seen (1 length of 40 fields)*	
		200x	*400x*
1+	36 (65.5)	30-299 AFB per 1 length	1-19 AFB per 1 length
2+	15 (27.2)	10-100 AFB per 1 field on average	20-199 AFB per 1 length
3+	4 (7.3)	>100 AFB per field on average	5-50 AFB per 1 field (20 fields on average)

[a] these samples were used to determine the effect of RPF on time to detect Mtb (n=55)

Supplementary Figure-1: Classification of sputum samples and the number of samples used in various assays

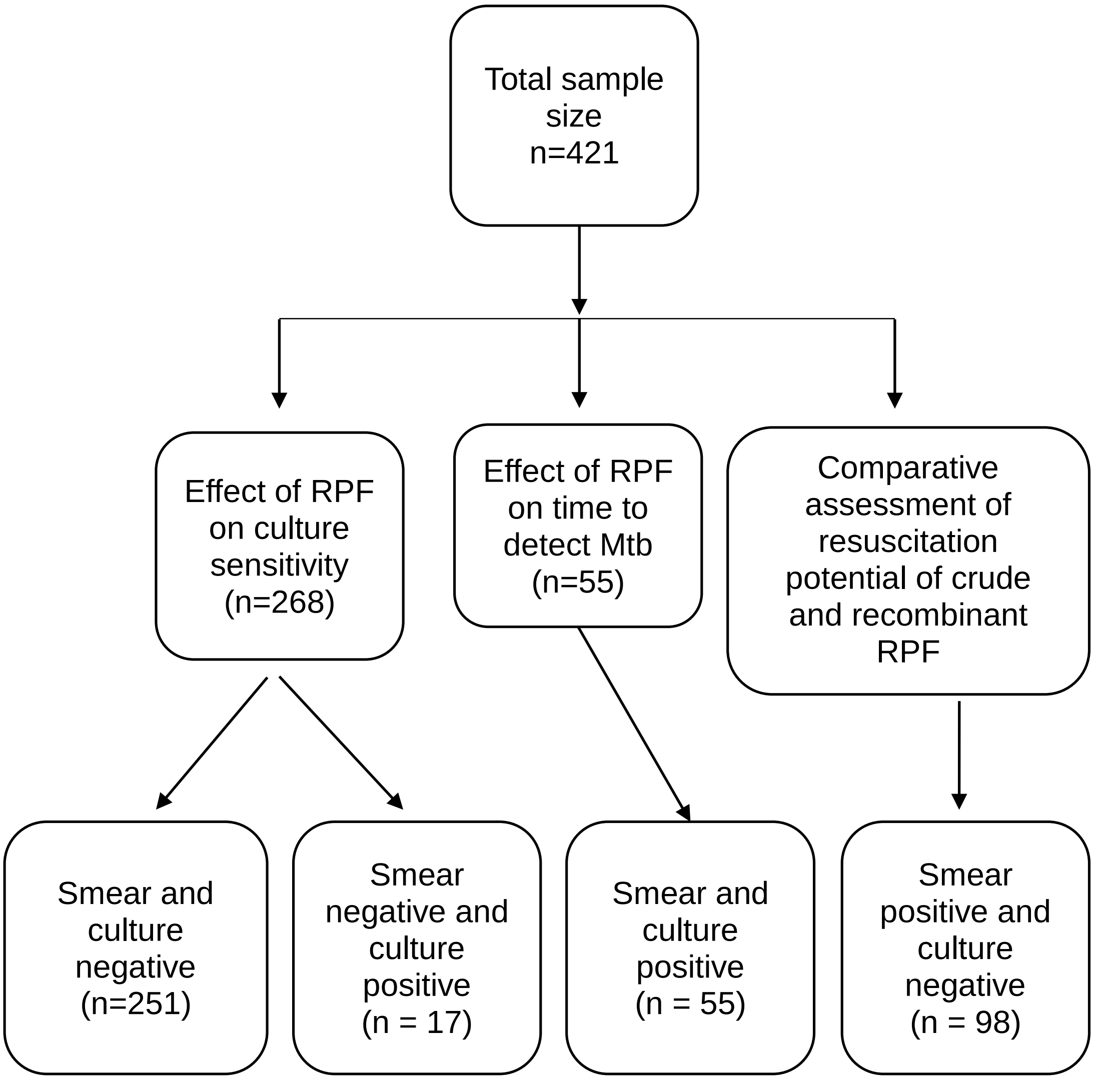

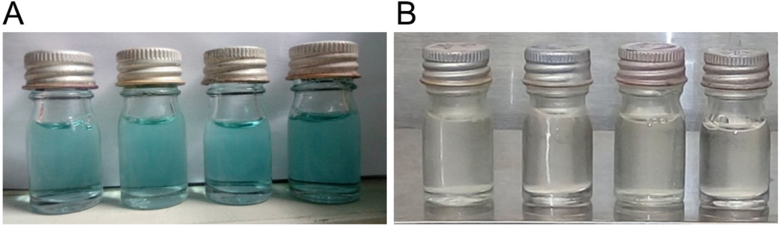
A
B

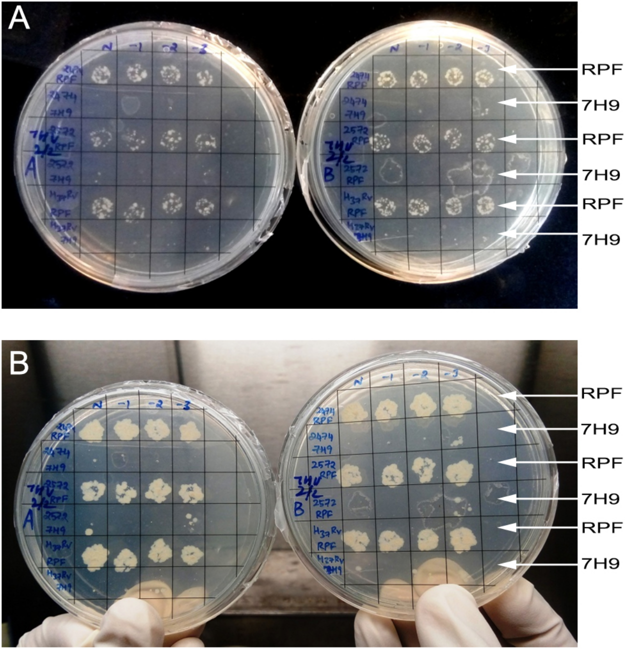
A
RPF
7H9
RPF
7H9
RPF
7H9
B
RPF
7H9
RPF
7H9
RPF
7H9
